# Shiftwork Is Associated with Higher Food Insecurity in U.S. Workers: Findings from a Cross-Sectional Study (NHANES)

**DOI:** 10.3390/ijerph19052847

**Published:** 2022-03-01

**Authors:** Maximilian Andreas Storz, Gianluca Rizzo, Mauro Lombardo

**Affiliations:** 1 Center for Complementary Medicine, Department of Internal Medicine II, Freiburg University Hospital, Faculty of Medicine, Univerity of Freiburg, 79106 Freiburg, Germany; 2 Independent Researcher, Via Venezuela 66, 98121 Messina, Italy; gianlucarizzo@email.it; 3 Department of Human Sciences and Promotion of the Quality of Life, San Raffaele Roma Open University, 00166 Rome, Italy; mauro.lombardo@uniroma5.it

**Keywords:** diet, nutrition, shiftwork, work schedule, consumer behavior, food security, eating habits, away-from-home meals, NHANES, food availability

## Abstract

The number of shift workers has increased substantially within the last decades to keep pace with the increasingly complex societal need for 24 h services. Shift work has been associated with unhealthy lifestyles and a lower overall diet quality. Little is known, however, with regard to food security and consumer behavior in shift workers. The present study sought to address this gap in the literature, exploring a sample of *n* = 4418 day workers and *n* = 1065 shift workers in the United States. Using cross-sectional data from the National Health and Nutrition Examination Surveys (NHANES, 2007–2010), we found that shiftwork was associated with a lower amount of money spent on eating out and higher food insecurity issues. Compared to day workers, a higher proportion of shift workers reported receipt of food stamps (12.5% vs. 23.4%, *p* < 0.001) and worried about running out of food (3.95% vs. 8.05%, *p* < 0.001). These associations remained significant after adjustment for confounders when using multivariate logistic regression. The number of not-home-prepared meals did not differ between both groups. In light of the population health disparities and adverse health outcomes associated with food insecurity, novel strategies are urgently warranted to improve the situation of shift workers.

## 1. Introduction

Traditionally, only a small proportion of the work force was engaged in shift work [1]. Due to growing economic pressures and to keep pace with the increasingly complex societal need for 24 h services, the number of shift workers has increased in many countries within the last decades [1,2,3,4]. This appears to be particularly true for the United States of America [5], where a significant proportion of the workforce works alternative hours [6].

Long atypical working hours and shift work have been associated with unhealthy lifestyle behaviors, such as smoking, drinking, physical inactivity, insufficient sleep, and impaired sleep quality [7,8,9]. Furthermore, both conditions have been associated with health-related productivity loss [10] and significantly increased odds for some chronic diseases and cancer [11,12].

One important factor that may partially explain these findings in shift workers is decreased diet quality [13]. Souza et al. recently reviewed the effects of shift work on diet quality and found a higher consumption of unhealthy foods in this group [14]. Shift workers tended to skip meals more frequently and also consumed more food at unconventional times. Hornzee at al. highlighted lower diet quality scores in shift workers (as compared to day workers), with a higher consumption of sugar-sweetened beverages and a more frequent usage of vending machines [15]. Although some studies could not confirm these findings (or even demonstrated opposite results [16]), it is now widely accepted that shift work is an important risk factor for a lower overall diet quality [17,18,19].

We hypothesized that this could be partially explained by higher food insecurity in shift workers (e.g., having limited reliable access to a sufficient quantity of affordable, nutritious food [20]). Lower wages and frequent changes in the work schedule could also predispose shift workers to unhealthy lifestyle behaviors, such as frequently eating meals prepared away from home (which has been associated with an increased all-cause mortality [21]).

Although conceivable, investigations on these potential associations are still scarce, and food security in shift workers has rarely been investigated in the literature. We sought to address this gap in the literature with our present study. The major aims of this cross-sectional analysis were twofold: (1) to investigate consumer behavior in shift workers, and (2) to obtain a better understanding of food security status in this group.

## 2. Materials and Methods

### 2.1. Study Design

We performed a cross-sectional study using aggregated data from the National Health and Nutrition Examination Survey (NHANES) [22]. The National Health and Nutrition Examination Survey (NHANES) is a large, biennial, stratified, multistage survey conducted by the Centers for Disease Control (CDC) [22,23]. One of the primary goals of the survey was to collect health and nutrition data on the US population. The NHANES is one of the largest and most important cross-sectional studies in terms of participant size, scope, ethical diversity, and free data accessibility [24]. Scientists worldwide have used NHANES data in the past to examine health-related questions in shift-working populations [25,26,27,28]. As a corollary, NHANES has contributed extensively to the assessment of potential associations between nutrition and numerous health outcomes in shift workers [25].

For the current analysis, we combined adult data from two different NHANES cycles (2007–2008 and 2009–2010) [29,30]. Approximately 5000 individuals participate in the NHANES annually [22,31]. All NHANES participants gave oral and written informed consent, and the study protocol was approved by the NCHS Research Ethics Review Board [32]. The NHANES data is publicly available from the Centers for Disease Control [22] and can be broadly categorized into six areas: demographics, examination, dietary, questionnaire, laboratory, and limited access [24].

### 2.2. Study Population, Outcome, and Exposure

For this study, we used data from various modules, including demographic, examination, and questionnaire data. We explain usage of particular data subsets hereafter. Shiftwork status was assessed using data from the Occupation Questionnaire Section (OQC) [33,34]. This module contains data on employment and other important variables relating to the daily work environment. The OQC included one question (OCQ265) entitled: “which of the following best describes the hours you usually work at your main job or business?” Shiftwork status was assessed based on this question with the following (potential) answers: (1) a regular daytime schedule, (2) a regular evening shift, (3) a regular night shift, (4) a rotating shift, and (5) another schedule. Answer 5 was not specified further, and thus removed for a lack of additional information [35]. We combined evening/night shift workers and rotating shift workers into one group (hereafter named shift workers). This group was compared to individuals on a regular daytime schedule. The 2007/2008 and 2009/2010 cycles were chosen because both included the aforementioned work schedule question, which has unfortunately been removed in later NHANES cycles [36].

We described the employed assessment of dietary behavior, food security, and consumer behavior in great detail in one of our previous publications [31]. In brief, we included data from three major modules: (a) the consumer behavior module, (b) the diet and nutrition behavior module, and (c) the food security module.

The Consumer Behavior (CBQ) section provided personal interview data on various diet-related consumer behavior topics [37]. This module was developed in partnership with the Economic Research Service of the U.S. Department of Agriculture [38]. It was introduced with NHANES 2007–2008 and comprised questions with regard to food expenditures, availability of certain type of foods in the family, as well as time spent on food shopping and cooking dinner [37,38]. The CBQ section also inquired about individual participant’s food choices (e.g., getting meals prepared away from home, meals obtained from fast-food or pizza places, usage of ready-to-eat meals and convenience food items bought in stores).

Among other things, NHANES participants were asked how often they had a certain type of food at their home [17]. Food items comprised salty snacks (such as chips and crackers), soft drinks (including fruit punch or fruit-flavored drinks), fruits (including frozen and canned fruits), and dark green vegetables. Answer categories included “never”, “rarely”, “sometimes”, “most of the time”, and “always”.

Two food expenditure questions inquired about the money spent on foods in the 30 days prior to the interview. Participants were given the opportunity to report the amount of money in USD as either per month or per week. All released variables were edited in order to standardize the reported amount to number of dollars in the last 30 days. Both NHANES cycles also included a question on the frequency of major food shopping, with six possible answers. These included “more than once a week”, “once a week”, “once every two weeks”, “once a month or less”, “rarely make major shopping trips”, “rarely shop for foods”.

The so-called Consumer Behavior Follow-up Module, a phone follow-up section to the CBQ that was introduced with NHANES 2007–2008 [39], was not included in the present analysis. The aim of this module was to gain additional insights into consumer behavior of NHANES participants. This module focused on factors that influence decisions to eat out and attitudes about changing current diets in NHANES participants. This section, however, was only answered by a subset of NHANES participants. As such, we refrained from adding this module to maintain an adequate sample size.

Another important component of the present analysis was three items from the Diet Behavior and Nutrition (DBQ) section [40]. The first questions inquired about the number of meals not prepared at home during the past 7 days (e.g., meals purchased in restaurants or fast-food places or obtained from vending machines). Two additional questions asked for the number of frozen meals/pizza and for the number of ready-to-eat foods eaten in the past 30 days. Ready-to-eat foods included soups, chicken, and sandwiches. When answering the second question, participants were explicitly instructed to exclude sliced meat or cheese bought for sandwiches and frozen or canned foods.

Finally, we also included four items from the Food Security (FSQ) section, which provided personal interview data on household and individual food security [41,42]. This section included binary questions (e.g., have {you/you or anyone in your household} ever received food stamp benefits?) and questions with multiple response categories. These inquired whether participants worried about running out of food within the last 12 months, whether bought food didn’t last, and whether participants couldn’t afford to eat balanced meals. In light of the high number of missing values for many FSQ variables [41,42], we only added the four particular aforementioned questions to maintain an adequate sample size.

We obtained participants’ demographic data from the demographics public release file [43]. Demographic data included sex, age, marital status, race/ethnicity, education level, and household income. Pre-defined variables and categories were not recoded, with the exception of marital status and household income. Marital status comprised the following categories: married or living with a partner, widowed/divorced/separated, and never married. Annual household income comprised two categories: over $20,000 and under $20,000, following the approaches of Muennig et al. and Hassoon et al. [44,45]. Examination data was limited to body mass index (BMI) [46], and categorized into four groups including obesity (BMI ≥ 30 kg/m^2^), overweight (BMI 25–29.99 kg/m^2^), normal weight (BMI 18.5–24.99 kg/m^2^), and underweight (BMI ≤ 18.49 kg/m^2^).

Smoking status was categorized according to a common method presented previously [47]. Sleep duration (in hours) was obtained from the Sleep Disorders (SLQ) module [48].

### 2.3. Statistical Analysis

We used sample weights provided by the NHANES to account for the complex survey design and oversampling in some populations during the two survey cycles. All analyses incorporated the primary sampling unit variable, the stratum variable, and the weighting variable. The weighting of NHANES data allows for extrapolation of study findings to the U.S. national population [49]. The interview weighting factor was divided by two for analyses using combined survey cycles.

We compared demographic, anthropometric, and other survey characteristics between shift workers and day workers. For our analysis, we used STATA 14 statistical software (StataCorp. 2015. Stata Statistical Software: Release 14. StataCorp LP, College Station, TX, USA). All continuous variables were compared by using appropriate sample weights for two-sample Student’s *t*-tests. All categorical variables were compared using STATA’s design-adjusted Rao–Scott test (a design-adjusted version of the Pearson chi-square test). When we found significant associations between shiftwork status and a particular outcome variable (e.g., lower fruit availability at home in shift workers), we re-investigated those associations using multivariate linear regression and logistic regression models adjusting for age, gender, race/ethnicity, education level, and annual household income. These covariates were identified as potential confounders because shift workers are often paid less in the labor market [50] and have lower educational attainment and income [10,51].

Statistical significance was determined at α = 0.05, and all tests for statistical significance were two-sided. Based on West’s applied survey data analysis recommendations, we preferred unconditional subclass analyses to preserve the main survey design and to provide larger standard errors [52].

Normally distributed variables were described with their mean and standard error in parentheses (see below). For categorical variables, we reported number of observations (*n*) as well as weighted proportions (and the corresponding standard error) in parentheses. For multivariate logistic regression models, we present odds ratios (OR) and the corresponding 95% confidence intervals (CIs).

## 3. Results

**Figure 1:**
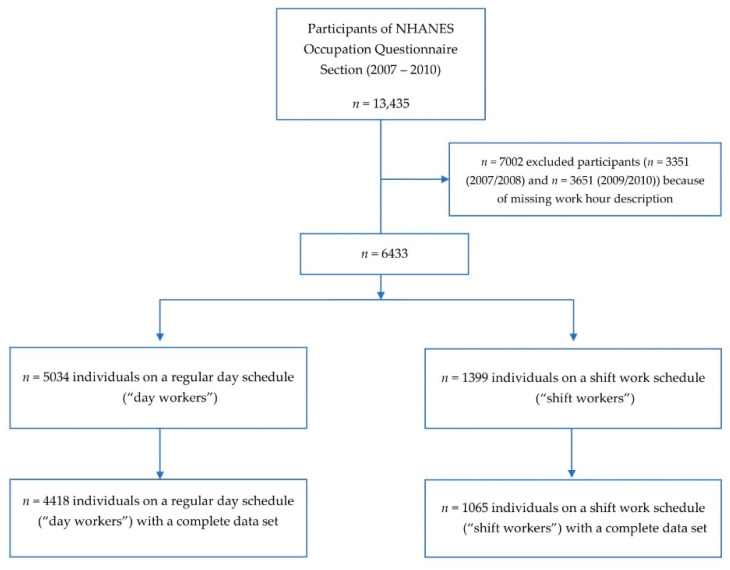
Participant inclusion flow chart: NHANES 2007–2010.

Between 2007 and 2010, *n* = 13,435 participants completed the NHANES Occupation Questionnaire section [33,34]. We excluded *n* = 7002 participants (*n* = 3351 (2007/2008) plus *n* = 3651 (2009/2010)) for missing or inconclusive work hour descriptions (Figure 1). As described in the methods, this step included removal of the category “another schedule” for lack of additional information (*n* = 538). The final sample included *n* = 5483 non-institutionalized participants after exclusion of all individuals with an incomplete data set. The sample comprised *n* = 4418 day workers and *n* = 1065 shift workers (Figure 1), which may be extrapolated to represent approximately 116,209,204 U.S. workers.

We present demographic, anthropometric, and other key characteristics of the study population in Table 1.

We found no significant intergroup differences with regard to gender. Our data, however, suggested a significant association between ethnicity/race and shiftwork status. The proportion of Hispanics and non-Hispanic Blacks was significantly higher in shift workers as compared to day workers. Non-Hispanic Whites comprised ‘only’ 59.73% (weighted) of shift workers, as opposed to more than 71% (weighted) in day workers. The proportion of never married shift workers was almost twice as high as in day workers (weighted).

We also observed a significant association between shiftwork status and education level. The proportion of individuals with a college degree (or higher) was significantly smaller among shift workers. In addition, we observed significant associations between shiftwork status and smoking status. The weighted proportion of current smokers was more than 8% higher in shift workers as in dayworkers.

Of note, our data suggested no significant intergroup differences with regard to body weight. The weighted proportion of obese shift-working participants tended to be higher; however, results were not significant. Day workers slept significantly longer than shift workers (6.82 h vs. 6.62 h per night, *p* < 0.001).

Table 2 displays the results of our food group availability analysis. We observed significant associations between shiftwork status and fruit and soft drink availability. The weighted proportion of day workers “always” having fruits and dark green vegetables at home was significantly higher as compared to shift workers. In contrast, we observed a higher proportion of individuals “rarely” or “never” having those items at home.

No significant intergroup differences were found with regard to salty snack availability. Finally, our results suggested a significantly lower availability of soft drinks at home in day workers. When we re-examined the aforementioned significant associations using multivariate logistic regression models (adjusting for age, gender, race/ethnicity, education level, and annual household income), those associations were no longer significant.

Table 3 compares consumer behavior between both groups. Shift workers consumed more frozen meals and pizza; however, the differences were not statistically significant. In addition to that, we did not discover significant intergroup differences with regard to the number of not-home-prepared meals. Day workers spent significantly more money (in USD) in supermarkets and grocery stores (USD 446.04 vs. USD 389.93, *p* = 0.011). The difference amounted to almost USD 60 per month. A comparable trend was observed with regard to money spent on eating out (*p* < 0.001). We re-analyzed both associations using multivariate linear regression models (Table 4) adjusting for various confounders. After adjustment for covariates, shiftwork status was no longer significantly associated with money spent at supermarket/grocery stores. The associations between shiftwork status and money spent on eating out remained significant after adjustment for confounders. A significant regression equation was found (F(16,17) = 13.13, *p* < 0.001), with an R² of 0.0625. Shift working significantly decreased the money spent on eating out in the last 30 days by USD −20.12 (CI: −38.11–(−2.13), *p* = 0.030) after adjusting for covariates including income. Larger coefficients were found for other socioeconomic variables in the model, including annual household income and educational level.

In addition to that, we observed no differences regarding “money spent on food at other stores” and regarding “money spent on carryout/delivered foods” (Table 3).

Shiftwork was associated with a lower shopping frequency in general. The (weighted) proportion of individuals shopping at least once a week was significantly lower in shift workers as opposed to day workers. In a multivariate logistic regression model adjusting for all covariates (not shown), this association was no longer significant.

**Figure 2:**
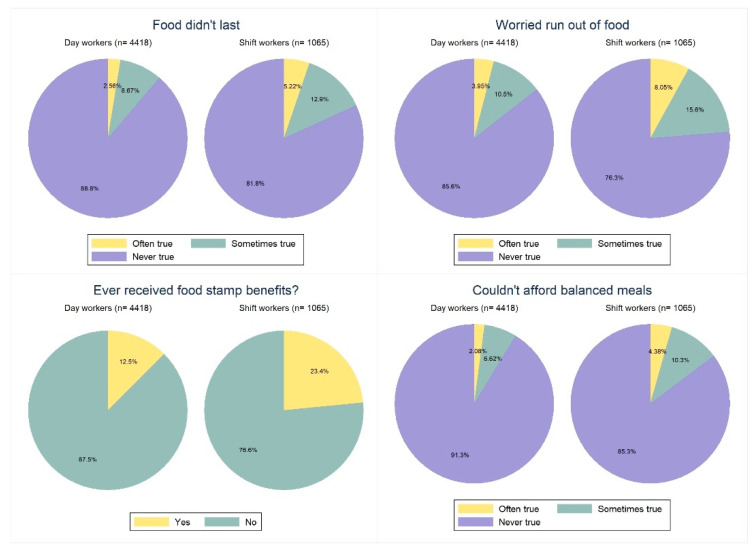
Food security in shift workers and day workers: a comparison regarding food stamp receipt, food concerns, affordability, and food sufficiency.

Figure 2 display the results of our food security analysis. A statistically significantly higher percentage of shift workers agreed with the statement that their food did not last (“often true” 5.22% vs. 2.56%, *p* < 0.001). The number of shift workers that reported receipt of food stamps was almost twice as high as in day workers (23.4% vs. 12.5%, *p* < 0.001), and shiftwork status was significantly associated with food stamp receipt (*p* < 0.001). Our results also suggest that a significantly higher proportion of shift workers worried about running out of food (Figure 2, *p* < 0.001). Moreover, affordability of balanced meals was significantly more problematic in shift workers as compared to day workers (*p* < 0.001).

We used multivariate logistic regression to re-examine all previously significant associations, adjusting for covariates (Table 5). The OR for receipt of food stamps was 1.44 (CI: 1.14–1.83) in shift workers (*p* = 0.004). For the other three food security items, we used the “often true” statement as the outcome variable (as compared to the combination of the other two categories, which served as the reference category). The OR for often “worrying run out of food” was 1.38 (CI: 1.02–1.87) in shift workers (*p* = 0.032) after adjusting for covariates. The OR that “food didn’t last” was 1.35 (CI: 1.01–1.81) in shift workers (*p* = 0.046) after adjusting for covariates. The association between shift work and a lower affordability of balanced meals was no longer significant in the employed logistic regression model (OR 1.45 (CI: 0.91–2.29), *p* = 0.108). OR for the other covariates are displayed in Table 5.

## 4. Discussion

We used cross-sectional data from the NHANES (2007–2010) to investigate consumer behavior and food security status in U.S. shift workers. Our data suggested significant associations between shiftwork and fruit and soft drink availability at home. Compared to shift workers, day workers spent significantly more money in supermarkets and grocery stores. After adjustment for potential confounders, these associations were no longer significant. Shiftwork was associated with substantial food security issues. These associations remained significant after adjustment for confounders. Of note, we found no significant intergroup differences with regard to the number of not-home-prepared meals.

Our results warrant a thorough discussion in the context of previous studies. Shift working has been associated with abnormal eating patterns [53], consumption of foods at more unconventional times [14], and a higher frequency of food cravings [54]. As such, it is not inconceivable that shiftwork increases frequency of eating meals prepared away from home.

Using data from the NHANES, Du and colleagues recently reported a significantly higher hazard ratio of mortality among individuals who ate meals prepared away from home very frequently (two meals or more per day), compared with those who seldom ate meals prepared away from home (fewer than one meal/wk) [21]. Hazard ratios were 1.49 (95% CI 1.05 to 2.13) for all-cause mortality, 1.18 (95% CI 0.55 to 2.55) for cardiovascular mortality, and 1.67 (95% CI 0.87 to 3.21) for cancer mortality. Our results suggest that the number of not-home-prepared meals is not higher in shift workers as compared to day workers. Our results revealed that shift workers spent significantly less money on eating out (USD 143.55 (7.53) vs. USD 186.17 (7.34)) than day workers, and this association remained significant after adjustment for confounders. One potential explanation for the non-significant intergroup differences in not-home-prepared meals might be that eating food out (at a restaurant or diner) or ordering food for takeout is very popular in the U.S. [55], and that the vast majority of Americans do not like to cook [56]. Based on a 2017 survey, only 10% of Americans like cooking [56], and as such it might be difficult to detect significant intergroup differences in our setting. While our findings might be seen as positive with regard to the results by Du and colleagues [21], we are less optimistic with regard to our food group availability analyses and food security analyses.

In 2015, Hemiö et al. reported that European male shift workers were less likely to consume vegetables (*p* < 0.001) and fruits (*p* = 0.049) on a daily basis than male day workers [57]. Our food group availability analysis revealed comparable findings, suggesting that U.S. shift workers have significantly less fresh fruit available at home (Table 2). In contrast, availability of soft drinks was significantly higher in this cohort; a result that confirms previous findings by Hornzee et al. [15]. We acknowledge that causal interference is impossible using cross-sectional data, and that after adjustment for confounders, those associations remained no longer significant. Lower wages and lower educational attainment associated with shift work may substantially influence dietary behavior and food choices in this cohort [10,50].

Earlier studies demonstrated that shift work has a considerable negative impact on diet quality [58]. Previous studies showed a substantial reduction in fiber intake in shift workers [59], as well as decreased intake in several micronutrients, such as vitamins A, D, and E, and zinc [60]. A regular intake of fresh, unprocessed plant foods (fruits, vegetables, legumes, nuts, and seeds) is essential to human health [61]. Our results, however, suggested a significantly lower shopping frequency in shift workers (that was no longer significant after adjustment for confounders). Only about 50% of the shift-working population went shopping on a weekly basis (whether due to low income or lack of time was not ascertainable from our cross-sectional data). This makes it difficult to guarantee a steady supply of fresh fruits and vegetables. It is conceivable that individuals shopping less frequently tend to rely on nonperishable and highly processed food options that tend to be high in saturated fat, sugar, and salt [62]. The fact that many shift workers reported food security problems (Figure 2) reinforces the hypothesis of limited access to fresh foods in this cohort.

Food security is a major problem in the United States and up to 10.5% of U.S. households suffered from it in 2020 [63]. Inequities in food security are a persistent point of concern in cities across the United States [64] and have a clear implication for population health disparities [65]. Consequences of food insecurity include (but are not limited to) mental health problems [66], physical health problems [67], and higher rates of chronic diseases [68]. Our results suggest that food insecurity appears to be significantly more prevalent among shift workers as compared to day workers (Figure 2). Shift work, for instance, significantly increased the OR for receipt of food stamps (OR: 1.44 (CI: 1.13–1.83) in shift workers.

A large proportion of shift workers indicated that their food did not last. One factor that may contribute to this phenomenon is the lower annual household income in that particular group (Table 2). Of note, the OR that “food did not last” remained significantly higher in shift workers even after adjustments for covariates.

Unrelated to our study data, low income has been identified as the factor that most negatively affected food security during the ongoing COVID-19 pandemic [69]. As a consequence, food security remains a current topic in the United States, and the COVID-19 pandemic (and its imposed social isolation) exacerbated persistent sociopolitical barriers to food security [64].

Unrelated to the COVID-19 pandemic, our data suggest that food security appears to be a problem in shift workers. As such, it is our hope to raise awareness for this issue with our study.

Future studies may address this and should investigate how food security can be improved under pandemic conditions. As such, well-planned intervention studies are urgently warranted.

### Limitations

This study has several strengths and limitations that warrant further discussion. Major strengths include the large and nationally representative dataset (National Health and Nutrition Examination Survey) as well as the largely unexplored study field (food security and consumer behavior in U.S. shift workers). Our modest sample size allowed for additional insights into shift worker’s consumer behavior and revealed new information that could serve as a basis for future studies. An additional strength of our approach is the representation of minorities in proportion to their representation in the general U.S. population [35].

Weaknesses include the cross-sectional nature of our data (which does not allow for causal inferences) and the use of self-report questions for shiftwork ascertainment. Unlike initially planned, we had to refrain from including the NHANES “consumer behavior phone follow-up module” in our study. Adding this subset of parameters would have substantially decreased the number of eligible cases with a complete dataset. After adjustment for potential confounders (which included age, gender, ethnicity, education level, and income), several associations were no longer significant. As such, it must be emphasized that it is difficult to disentangle the complex interactions between shiftwork, income, education, and nutrition using cross-sectional data. We also acknowledge that our data dates back to 2007–2010. Unfortunately, newer NHANES cycles did not include the key occupation variables employed in this study, and as such, we were unable to present more current data. Given that the current (2020) food insecurity prevalence is the United States is comparable to that in 2008–2010 [63], we believe that our data is still worth reporting. Aside from that, few studies have looked at food security in shift workers, which seem to be at a particular risk according to our results.

## 5. Conclusions

Shift workers are more likely to be affected by food insecurity as compared to their day-working counterparts. Those associations remained significant after adjusting for various confounders, including income and education level. In light of the population health disparities and the numerous adverse health outcomes associated with food insecurity, the authors call for novel strategies to improve the overall situation of shift workers.

## Figures and Tables

**Table 1:** Study sample characteristics: a comparison by shiftwork status.

	Day Workers (*n* = 4418)	Shift Workers (*n* = 1065)	*p*-Value
Sex			
Male	*n* = 2372 (53.73% (0.86))	*n* = 556 (52.20% (1.8))	0.476
Female	*n* = 2046 (46.27% (0.86))	*n* = 509 (47.80% (1.8))	
Race/ethnicity			
Mexican American	*n* = 898 (8.96% (1.33))	*n* = 209 (9.97% (1.45))	<0.001
Other Hispanic	*n* = 500 (4.85% (0.80))	*n* = 140 (7.08% (1.45)) ^a^	
Non-Hispanic White	*n* = 2075 (71.05% (2.47))	*n* = 385 (59.73% (3.13)) ^a^	
Non-Hispanic Black	*n* = 748 (9.13% (0.99))	*n* = 278 (16.85% (1.76)) ^a^	
Other race	*n* = 197 (6.02% (0.8))	*n* = 53 (6.36% (1.05))	
Marital status			
Married/living with partner	*n* = 2947 (69.37% (1.06))	*n* = 546 (52.65% (2.14)) ^a^	<0.001
Widowed/divorced/separated	*n* = 719 (14.01% (0.66))	*n* = 199 (16.00% (1.26))	
Never married	*n* = 752 (16.62% (0.94))	*n* = 320 (31.35% (1.51)) ^a^	
Education level			
Less than 9th grade	*n* = 390 (4.1% (0.46))	*n* = 107 (5.19% (0.58))	<0.001
9–11th grade	*n* = 617 (10.43% (0.72))	*n* = 172 (13.62% (1.39)) ^a^	
High school grad/GED	*n* = 999 (22.05% (1.06))	*n* = 288 (28.84% (1.73)) ^a^	
Some college or AA degree	*n* = 1219 (28.79% (0.92))	*n* = 363 (36.84% (1.83)) ^a^	
College graduate or above	*n* = 1193 (34.63% (1.60))	*n* = 135 (15.53% (1.13)) ^a^	
Annual household income			
Under $20,000	*n* = 530 (7.46% (0.58))	*n* = 203 (15.1% (0.89)) ^a^	<0.001
Over $20,000	*n* = 3888 (92.54% (0.58))	*n* = 862 (84.90% (0.88)) ^a^	
Smoking status			
Never smoker	*n* = 2557 (57.66% (1.33))	*n* = 598 (54.65% (2.46))	<0.001
Former smoker	*n* = 934 (22.12% (1.12))	*n* = 171 (16.86% (2.25)) ^a^	
Current smoker	*n* = 927 (20.22% (0.75))	*n* = 296 (28.49% (1.93)) ^a^	
Body Weight			
Underweight	*n* = 54 (1.5% (0.24))	*n* = 13 (1.32% (0.38))	0.313
Normal weight	*n* = 1180 (28.61% (0.88))	*n* = 285 (29.84% (2.28))	
Overweight	*n* = 1528 (35.14% (1.15))	*n* = 334 (31.11% (2.19))	
Obesity	*n* = 1656 (34.74% (1.03))	*n* = 433 (37.73% (2.43))	
Age			
Mean (SE)	43.04 (0.32)	37.89 (0.40)	<0.001
Sleep duration (hours)			
Mean (SE)	6.82 (0.02)	6.62 (0.06)	<0.001

Legend: Column percentages may not equal 100% due to rounding. The *p*-value is based on STATA’s design-based Rao–Scott F-test and tests for a potential association between shiftwork status and the respective variable (categorical variables only). ^a^: indicates significant differences in the weighted proportions.

**Table 2:** Food availability at home: a comparison by shiftwork status.

	Day Workers (*n* = 4418)	Shift Workers (*n* = 1065)	*p*-Value
Soft drinks available at home			
Always	*n* = 1718 (40.08% (1.43))	*n* = 438 (43.92% (2.31))	0.036
Most of the time	*n* = 642 (13.93% (0.72))	*n* = 193 (16.45% (1.28))	
Sometimes	*n* = 815 (16.69% (0.87))	*n* = 187 (15.22% (1.55))	
Rarely	*n* = 624 (14.83% (1.01))	*n* = 118 (11.78% (1.20)) ^a^	
Never	*n* = 619 (14.47% (0.67))	*n* = 129 (12.63% (1.41))	
Salty snacks available at home			
Always	*n* = 1729 (42.06% (1.55))	*n* = 409 (42.66% (1.96))	0.072
Most of the time	*n* = 870 (21.34% (0.88))	*n* = 196 (20.04% (1.64))	
Sometimes	*n* = 1126 (23.45% (1.25))	*n* = 289 (22.99% (1.31))	
Rarely	*n* = 522 (10.27% (0.81))	*n* = 119 (10.62% (1.36))	
Never	*n* = 171 (2.88% (0.28))	*n* = 52 (3.68% (0.68))	
Dark green vegetables available at home			
Always	*n* = 2438 (54.66% (1.45))	*n* = 532 (49.46% (2.32)) ^a^	0.095
Most of the time	*n* = 1005 (23.17% (1.06))	*n* = 253 (23.97% (1.46))	
Sometimes	*n* = 690 (15.22% (1.01))	*n* = 189 (17.48% (1.24))	
Rarely	*n* = 191 (4.66% (0.44))	*n* = 66 (6.14% (0.94))	
Never	*n* = 94 (2.29% (0.45))	*n* = 25 (2.94% (0.75))	
Fruits available at home			
Always	*n* = 2931 (67.61% (1.18))	*n* = 629 (60.06% (2.38)) ^a^	0.006
Most of the time	*n* = 835 (18.33% (0.98))	*n* = 241 (23.11% (2.12)) ^a^	
Sometimes	*n* = 489 (10.32% (0.69))	*n* = 132 (10.70% (1.16))	
Rarely	*n* = 138 (3.16% (0.36))	*n* = 52 (5.11% (1.09))	
Never	*n* = 25 (0.58% (0.16))	*n* = 11 (1.02% (0.36))	

Legend: Column percentages may not equal 100% due to rounding. The *p*-value is based on STATA’s design-based Rao–Scott F-test and tests for a potential association between shiftwork status and availability of a certain food group. ^a^: indicates significant differences in the weighted proportions.

**Table 3:** Consumer behavior in day workers and shift workers: an overview.

	**Day Workers** **(*n* = 4418)**	**Shift Workers** **(*n* = 1065)**	***p*** **-Value**
Home cooking/eating habits			
# of times someone cooked dinner at home	4.89 (0.05)	6.92 (2.30)	0.380
# of meals not home prepared	4.39 (0.09)	4.62 (0.17)	0.226
# of ready-to-eat foods in past 30 days	1.92 (0.11)	2.09 (0.16)	0.421
# of frozen meals/pizza in past 30 days	2.74 (0.11)	3.21 (0.28)	0.099
Food expenditures			
Money spent at supermarket/grocery store (USD)	446.04 (17.15)	389.93 (13.56)	0.011
Money spent on food at other stores (USD)	63.08 (3.24)	61.81 (3.96)	0.747
Money spent on eating out (USD)	186.17 (7.34)	143.55 (7.53)	<0.001
Money spent on carryout/delivered foods (USD)	27.93 (1.73)	26.75 (2.37)	0.590
Frequency of major food shopping			
More than once a week	*n* = 568 (12.21% (0.96))	*n* = 121 (10.4% (1.35)) ^a^	<0.001
Once a week	*n* = 2132 (50.80% (1.09))	*n* = 448 (42.53% (2.08)) ^a^	
Once every two weeks	*n* = 1140 (25.54% (0.91))	*n* = 319 (32.40% (1.81)) ^a^	
Once a month or less	*n* = 492 (9.84% (0.62))	*n* = 154 (12.75% (1.02))	
Rarely make major shopping trips	*n* = 67 (1.38% (0.28))	*n* = 14 (1.39% (0.40))	
Rarely shop for foods	*n* = 19 (0.32% (0.09))	*n* = 9 (0.51% (0.16))	

Legend: Column percentages may not equal 100% due to rounding. The *p*-value for categorical variables is based on STATA’s design-based Rao–Scott F-test and tests for a potential association between shiftwork status and frequency of major food shopping. ^a^: indicates significant differences in the weighted proportions.

**Table 4:** Linear regression models investigating associations of shiftwork status and (1) money spent in supermarkets/grocery stores and (2) on eating out in the past 30 days.

	Money Spent at Grocery Stores/Supermarkets	*p*	Money Spent on Eating Out	*p*
Gender				
Female	−29.69 (−74.64–15.26)	0.188	−21.95 (−34.13–(−9.77))	0.001
Male	-		-	
Age				
18–24 years	-		-	
25–34 years	51.81 (−67.47–171.10)	0.383	−21.27 (−53.61–11.06)	0.190
35–44 years	44.05 (−3.85–91.97)	0.070	3.83 (−33.42–41.10)	0.835
45–54 years	46.26 (6.87–85.60)	0.023	−20.10 (−49.88–9.67)	0.179
55–64 years	−17.23 (−69.55–35.07)	0.507	−30.96 (−64.62–2.68)	0.070
>65 years	−61.43 (−120.76–(−2.11))	0.043	−40.26 (−88.46–7.94)	0.099
Ethnicity				
Mexican American	−21.53 (−126.57–83.50)	0.679	−12.86 (−32.18–6.46)	0.185
Other Hispanic	−2.12 (−85.92–81.66)	0.959	−18.50 (−41.47–4.47)	0.111
Non-Hispanic White	-		-	
Non-Hispanic Black	−140.25 (−207.04–(−73.45)	<0.001	−60.38 (−72.80–(−47.96))	<0.001
Other race	−60.16 (−124.11–3.77)	0.064	−6.86 (−72.47–58.83)	0.834
Education level				
Less than 9th grade	85.77 (3.66–167.88)	0.041	−101.45 (−139.91–(−62.99))	<0.001
9–11th grade	117.03 (−138.22–372.29)	0.357	−93.06 (−120.57–(−65.54))	<0.001
High school grad/GED	1.70 (−34.80–38.21)	0.925	−77.74 (−107.93–(−47.56))	<0.001
Some college or AA degree	−14.27 (−50.33–21.79)	0.426	−61.38 (−86.07–(−36.69))	<0.001
College graduate or above	-		-	
Annual household income				
Under $20,000	−98.87 (−168.34–(−29.41))	0.007	−76.59 (−91.22–(−61.95))	<0.001
Over $20,000	-		-	
Shiftwork status				
Day worker	-		-	
Shift worker	−40.12 (−85.67–5.42)	0.082	−20.12 (−38.11–(−2.13))	0.030

Legend: Coefficients are displayed with their 95% confidence intervals and *p*-value. The symbol “-” indicates the reference category. *p* = *p*-value.

**Table 5:** Logistic regression models investigating associations of shiftwork status and food security items.

	Food Stamps Receipt	*p*	Worried to Run Out of Food	*p*	Food Did Not Last	*p*	Food Affordability	*p*
Gender								
Female	1.23 (1.06–1.44)	0.008	1.37 (1.10–1.70)	0.005	1.06 (0.84–1.34)	0.599	1.19 (0.86–1.64)	0.262
Male	-		-		-		-	
Age								
18–24 years	-		-		-		-	
25–34 years	1.22 (0.95–1.55)	0.106	0.93 (0.59–1.45)	0.749	0.77 (0.45–1.30)	0.323	1.04 (0.58–1.87)	0.867
35–44 years	1.27 (0.90–1.79)	0.159	1.09 (0.66–1.79)	0.709	0.87 (0.45–1.68)	0.690	1.02 (0.52–1.99)	0.946
45–54 years	0.75 (0.54–1.04)	0.091	0.62 (0.35–1.10)	0.104	0.78 (0.40–1.51)	0.463	0.76 (0.36–1.58)	0.459
55–64 years	0.72 (0.48–1.09)	0.118	0.40 (0.23–0.71)	0.002	0.52 (0.25–1.10)	0.087	0.34 (0.16–0.70)	0.005
>65 years	0.40 (0.19–0.83)	0.016	0.15 (0.07–0.34)	<0.001	0.28 (0.11–0.70)	0.008	0.19 (0.06–0.59)	0.006
Ethnicity								
Mexican American	1.91 (1.25–2.92)	0.095	2.81 (1.77–4.43)	<0.001	3.92 (2.21–6.93)	<0.001	1.74 (0.78–3.87)	0.164
Other Hispanic	1.62 (1.03–2.53)	0.034	2.75 (1.74–4.33)	<0.001	4.22 (2.24–7.96)	<0.001	1.82 (0.81–4.05)	0.137
Non-Hispanic White	-		-		-		-	
Non-Hispanic Black	3.41 (2.34–4.96)	<0.001	2.60 (1.79–3.80)	<0.001	3.51 (2.19–5.61)	<0.001	1.72 (0.97–3.04)	0.061
Other race	1.14 (0.63–2.06)	0.640	1.19 (0.64–2.19)	0.564	1.92 (1.11–3.22)	0.020	1.48 (0.63–3.44)	0.346
Education level								
Less than 9th grade	3.80 (2.59–5.57)	<0.001	7.53 (3.50–16.2)	<0.001	9.87 (4.41–22.1)	<0.001	10.63 (3.2–35.31)	<0.001
9–11th grade	6.39 (4.79–8.53)	<0.001	5.89 (2.73–12.7)	<0.001	9.14 (4.18–19.9)	<0.001	9.02 (3.15–25.85)	<0.001
High school grad/GED	4.50 (3.36–6.02)	<0.001	4.57 (2.16–9.67)	<0.001	6.24 (3.02–12.9)	<0.001	6.78 (2.44–18.82)	0.001
Some college or AA degree	2.83 (1.99–4.01)	<0.001	3.53 (1.64–7.57)	0.002	4.27 (2.10–8.68)	<0.001	3.16 (1.14–8.80)	0.028
College graduate or above	-		-		-		-	
Annual household income								
Under $20,000	4.74 (3.55–6.33)	<0.001	3.73 (2.85–4.87)	<0.001	3.87 (2.56–5.86)	<0.001	2.96 (1.79–4.87)	<0.001
Over $20,000	-		-		-		-	
Shiftwork status								
Day worker	-		-		-		-	
Shift worker	1.44 (1.13–1.83)	0.004	1.38 (1.02–1.87)	0.032	1.35 (1.01–1.81)	0.046	1.45 (0.91–2.29)	0.108

Legend: OR are displayed with their 95% confidence intervals and *p*-value. The symbol “-” indicates the reference category. *p* = *p*-value.

## Data Availability

Data are publicly available online (https://wwwn.cdc.gov/nchs/nhanes/Default.aspx; accessed on 3 January 2022). The datasets used and analyzed during the current study are available from the corresponding author on reasonable request.
